# Editorial: Deciphering mechanisms of plant adaptation and resistance under cold temperature stress

**DOI:** 10.3389/fpls.2024.1460573

**Published:** 2024-08-19

**Authors:** Sabir Hussain Shah, John E. Carlson, Karl J. Niklas, Adalberto Benavides-Mendoza, Felipe Klein Ricachenevsky

**Affiliations:** ^1^ Department of Agricultural Sciences, Faculty of Sciences, Allama Iqbal Open University, Islamabad, Pakistan; ^2^ Department of Ecosystem Science and Management, Pennsylvania State University, University Park, PA, United States; ^3^ Plant Biology Section, School of Integrative Plant Science, Cornell University, Ithaca, NY, United States; ^4^ Department of Horticulture, Universidad Autónoma Agraria Antonio Narro, Saltillo, Mexico; ^5^ Botany Department, Institute of Biosciences, Federal University of Rio Grande do Sul, Porto Alegre, RS, Brazil; ^6^ Center of Biotechnology, Federal University of Rio Grande do Sul, Porto Alegre, RS, Brazil

**Keywords:** chilling, cold-acclimation, cold-responsive genes, signal-perception, transcriptional-regulation

## Introduction

Low, non-freezing temperatures pose a significant threat to crop species affecting their productivity, survival, and ecological distributions (Shah et al., 2017; Adhikari et al., 2022). Cold stress severely disrupts various physiological processes, including membrane fluidity, water and ionic balance, and the generation of reactive oxygen species (ROS) that impair DNA, RNA, and protein stability. Additionally, it hampers photosynthetic efficiency and retards biochemical reactions (Bailey-Serres et al., 2019). As sessile organisms, plants must adapt to adverse environmental conditions by evolving effective *in situ* survival mechanisms (Pareek et al., 2017). The adaptations to withstand low temperatures are collectively called “cold acclimation”. Temperatures that can affect the growth of plants vary among species and even among conspecific populations. For example, a cooler temperatures (<15°C) are sufficient to evoke cold injuries in several species of tropical and sub-tropical origins (Hincha and Zuther, 2014). Cold tolerance is defined broadly as the ability of plants to endure seasonal low but nonfreezing temperatures typically ranging from 0 to 15°C, whereas freezing tolerance refers to the ability to survive subzero temperatures (< 0°C). Cold acclimation primarily involves the modulation of gene expression and metabolic adjustments that can collectively result in a range of morphological, biochemical, and physiological changes (Fang et al., 2021; Guo et al., 2021).

The current Research Topic, ‘Deciphering mechanisms of plant adaptation and resistance under cold temperature stress’, includes six papers that address important aspects of plant adaptation to cold stress.

## Role of GRAS gene family in *Liriodendron chinense*

Weng et al. identified 49 members of the LcGRAS gene family in *Liriodendron chinense* employing a genome-wide approach. Their findings disclose a unique branch (HAM-t), adding to the understanding of the evolutionary dynamics within the GRAS gene family. The expression patterns of *LcGRAS* genes were explored during somatic embryogenesis, indicating their involvement during perhaps the most critical developmental episode in plant ontogeny. One of the major contributions of this research is the investigation of the response of *GRAS* genes to abiotic stress. The authors reveal the activation of genes in the PAT subfamily under stress conditions, thereby showing their potential role in mediating plant responses to environmental challenges. The observed modulation of PIF and COR expression following the transient overexpression of PATs provides the regulatory mechanisms underlying cold stress responses in *L. chinense*.

## Photosynthesis-related genes enhance tolerance against low temperature

Li et al. explored molecular mechanisms underlying photosynthetic regulation in *Lavandula angustifolia* Mill under cold stress. This study offers valuable insights into the adaptive strategies in this species, particularly in the harsh climatic conditions of Harbin, northeast China. The study elucidates the transcriptional responses of photosynthesis-related genes to varying temperatures by employing RNA-seq technology coupled with physiological measurements of photosynthetic parameters. The findings reveal that the identification of key genes, including *LaBAM1s*, *LaMPK4-1*, *LaMMK2*, *LaFBA*, *LaOMTs*, *LaGAPAs*, *LaGOX*, *LaTKL1s*, and *LaPSY* and their expression patterns in response to temperature fluctuations provide an increased understanding of the molecular basis of cold tolerance in *L. angustifolia*.

## Metabolism of nonstructural carbohydrates, lipids, and energy in *Cycas* species

Wu et al. reported the discovery of metabolic mechanisms of nonstructural carbohydrates (NSC), lipids, and energy governing tolerance to unexpected freezing stress in two *Cycas* species, *C. panzhihuaensis* and *C. bifida.* In this study, higher stability of photosynthetic machinery was observed in *C. panzhihuaensis*, highlighting its enhanced resilience to unexpected freezing events compared to *C. bifida*. The findings reveal that the dynamic changes in NSC and lipid profiles, including alterations in soluble sugars and glycerolipids, offer valuable insights into the metabolic adaptations of these species to freezing stress.

## Freezing tolerance in chickpeas

Kalve et al. unraveled the performance of eleven wild accessions of chickpea and two commercial cultivars, CDC Leader and CDC Consul along with a cold sensitive variety ILC533 as a check under freezing stress. In this study, the authors assessed changes in gene expression through transcriptome analysis using mRNA sequencing. The findings of this study reveal that CBF pathway-related genes were overexpressed during freezing stress in the wild relative Kesen_075, and overexpression of these genes alleviated the damage caused by freezing stress. QTL-seq analysis was also done to identify genomic regions associated with tolerance to freezing stress in an F2 generation that was obtained by crossing CDC Consul and Kesen_075. This QTL-seq analysis confirmed ten QTLs associated with freezing stress tolerance in chickpea.

## Role of ABA and ethylene in cold stress

Qian et al. revealed the crucial role of ABA and ethylene in the cold stress response of *Tetrastigma hemsleyanum*, which is a valuable herb utilized in modern medicine. In this study, a comprehensive transcriptome analysis was performed to investigate the molecular mechanisms during cold stress. Plant hormones were induced by short (2 h) and long (9 h) treatments of frost, and multiple transcripts and genes associated with the cold stress were identified. The findings demonstrate that the endogenous ABA and ethylene contents are increased following cold stress that enhanced cold tolerance in both frost sensitive and frost tolerant ecotypes.

## Mechanism of clonal growth in alpine perennials

Mishra et al. studied the molecular mechanisms governing adventitious rooting in Arctic alpine species under cold stress. This research used a combination of physiological studies, transcriptomics, and histological analyses that unveiled a complex gene regulatory network coordinating the formation of adventitious roots in Arabis alpine. The identification of specific internodes where adventitious roots develop and hormone responses at different stages of roots formation provides valuable insights into the molecular mechanisms of this adaptive process.

## Conclusion

The Research Topic of research papers in this editorial represents significant advancements in our understanding of plant adaptation and resistance to cold temperature stress, especially in non-model species. By deciphering the molecular mechanisms underlying several processes, researchers are paving the way for the development of novel strategies to enhance crop resilience and ensure food security in the face of changing climatic conditions. As we continue to unravel the complexities of plant responses to environmental stresses, the findings presented in this Research Topic will undoubtedly inform future efforts aimed at mitigating the impacts of cold stress on global agriculture and ecosystem health.

## Author contributions

SS: Writing – original draft. JC: Writing – review & editing. KN: Writing – review & editing. AB: Writing – review & editing. FR: Writing – review & editing.
